# Delay in health-care-seeking treatment among tuberculosis patients in Japan: what are the implications for control in the era of universal health coverage?

**DOI:** 10.5365/wpsar.2019.10.1.010

**Published:** 2020-06-30

**Authors:** Reina Yoshikawa, Lisa Kawatsu, Kazuhiro Uchimura, Akihiro Ohkado

**Affiliations:** a Department of General Internal Medicine, Rakuwakai Marutamachi Hospital, Kyoto, Japan.; b Department of Epidemiology and Clinical Research, the Research Institute of Tuberculosis, Japan Anti-Tuberculosis Association, Tokyo, Japan.; c Nagasaki University Graduate School of Biomedical Sciences, Nagasaki, Japan.

## Abstract

**Objective:**

To study the trends in and risk factors for patient delay (the time from the onset of symptoms to the initial doctor visit) in pulmonary tuberculosis (PTB) using three temporal categories − short (2 weeks to < 2 months), medium (2 months to < 6 months) and long (≥ 6 months) − and discuss implications for social protection measures.

**Methods:**

A descriptive cross-sectional study was conducted by analysing Japanese TB surveillance data from patients with symptomatic PTB registered between 2007 and 2017 (*n* = 88 351).

**Results:**

While the proportion of patients with short delay has decreased significantly (*P* < 0.001), the proportions of those with medium or long delays have decreased slightly (*P* = 0.0015 and *P* < 0.001, respectively). Not having health insurance, receiving public assistance, being a temporary worker, and having a history of homelessness were some of the risks identified for patient delay. Being male and working full-time were two risks specifically associated with long delay (for males, the adjusted odds ratio = 1.17, *P* < 0.05; for being a full-time worker, the adjusted odds ratio = 1.72, *P* < 0.05).

**Discussion:**

Despite the implementation of universal health coverage decades ago, patient delay remains a challenge in Japan. Our study identified various risk factors, many of which could have been resolved if appropriate social protection measures were in place, indicating shortcomings in universal health coverage in Japan and the need for continued effort to ensure that no one is left behind.

Tuberculosis (TB) continues to be a major global health issue, with 10 million people having newly diagnosed disease and 1.2 million dying from it in 2018. (*1*) The World Health Organization (WHO) developed the End TB Strategy in 2014, with three major targets to be achieved by 2035: a 90% reduction in TB incidence compared with 2015, a 95% reduction in TB deaths compared with 2015, and no affected families facing catastrophic financial losses from TB. (*2*) Early case detection is one of the key components of this strategy, not only to allow for early diagnosis and treatment, and thus better treatment outcomes for patients, but also to terminate the chain of transmission. (*3*) Yet previous studies have shown that delays on the part of the patient and the health system have continued to be unacceptably high, with factors such as unemployment and poverty playing major roles in affecting a delay in diagnosis. (*4*, *5*) Increasingly, it is recognized that policy efforts are needed to address these socioeconomic factors in line with the overarching framework for achieving universal health coverage (UHC). (*6*)

Japan introduced the first national policy for social health insurance in 1922. Later, in response to the call for welfare policies to mitigate social instability after Second World War, UHC was achieved in 1961 through the co-existence of different public health insurance schemes. (*7*) Additionally, Japan has maintained cost equality across schemes by regulating fee schedules and co-payment rates, with charges for elderly people and children being one third of those for other adults. (*7*) UHC ensures free access to any medical institution, and community-based health services are available at municipal public health centres, including TB screening for high-risk groups in the community. Today, Japanese people have access to one of the most affordable, high-quality and egalitarian health systems in the world. (*8*)

Yet, the rate of decrease in Japan’s TB notification rate has stagnated since the 1990s, reaching 13.3 cases/100 000 population in 2017, and the prospect of achieving the national target of less than 10 cases/100 000 by 2020 seems unlikely. (*9*) One of the possible issues lies with the time from the onset of symptoms of TB to the initial doctor visit, which is known as patient delay; while the proportion of patients with TB experiencing doctor delay, the time from the initial doctor visit to diagnosis, has remained relatively constant, the proportion with patient delay has been increasing during the past 20 years. (*9*) In 2017, the proportion of patients with symptomatic pulmonary TB (PTB) who took more than 2 months to access medical services after the onset of symptoms was as high as 20.0%. (*9*)

The objectives of this study were to conduct a detailed analysis of patient delay in Japan, investigate the risk factors for patient delay and discuss implications for social protection measures for TB patients, especially in a country where UHC was achieved decades ago.

## Methods

We conducted a cross-sectional study of symptomatic PTB patients, newly notified to the nationwide TB surveillance system, Japan Tuberculosis Surveillance (JTBS), between 1 January 2007 and 31 December 2017. In the current JTBS system, providing information regarding symptoms for all patients notified as having PTB is mandatory. A symptomatic PTB patient is defined as someone who has complained not only of respiratory but also of any other general symptoms.

### Japan Tuberculosis Surveillance system

Japan introduced its first nationwide computerized TB surveillance system in 1987. TB is a notifiable disease, and public health centres are responsible for collecting and entering data about notified patients into the system. Data items included in the JTBS system are sex, age, nationality, occupation, whether the patient has health insurance and what type, history of homelessness, history of treatment, symptoms, sputum smear result, presence of diabetes mellitus (DM), and delay information, including date of symptom onset and date of initial doctor visit. The data are summarized monthly and annually and are available online. Mechanisms to ensure data quality include the system’s automatic verification programme, as well as regular meetings attended by staff from hospitals and public health centres. Periodic refresher trainings on data entry are also provided to staff at public health centres across the nation.

### Definition of patient delay

Patient delay is defined in the JTBS system as the time between the date of symptoms onset and the initial doctor visit, and it is automatically calculated and categorized as < 2 weeks, ≥ 2 weeks to < 1 month, ≥ 1 month to < 2 months, ≥ 2 months to < 3 months, ≥ 3 months to < 6 months, ≥ 6 months, unknown, and not applicable. Previous studies in Japan have generally used a binary definition, with patient delay being defined as a delay of > 2 months between the onset of symptoms and the initial doctor visit and no delay defined as < 2 months. (*9*-*11*)

We first extracted data from all PTB patients who were registered as having symptoms, then re-categorized them into four definitions of delay: no delay, short delay (≥ 2 weeks to < 2 months), medium delay (≥ 2 months to < 6 months) and long delay (≥ 6 months).

### Data analysis and ethics

The numbers and proportions of symptomatic PTB patients in the three categories of delay were summarized, and trends were tested using the Cochran−Armitage test. The trends in the proportion of those in each delay category were also calculated by country of birth. More than 60% of Japan-born patients are elderly and they tend to present after a shorter delay, while the majority of younger patients are foreign born and they tend to present after a longer delay. (*9*) Due to this heterogeneity in age variance by country of birth, comparisons were made for all age groups combined and then repeated for those who were younger than 65 years.

Characteristics of patients with and without patient delay were summarized and proportions were compared; multinomial logistic regression analysis was conducted to identify possible risk factors for the three categories of patient delay. Risk factor variables were selected based on the associated factors identified in previous studies. (*8*-*11*)

R version 3.1.3 (R Development Core Team, Vienna, Austria) was used for all statistical analyses.

### Ethics statement

The study protocol was approved by the Institutional Review Board of the Research Institute of Tuberculosis, Japan Anti-Tuberculosis Association (reference no. RIT/IRB 30–9). Informed consent was deemed not necessary by the review board, as the surveillance data do not contain personal identifiers.

## Results

Between 2007 and 2017, a cumulative total of 134 869 symptomatic PTB patients were newly notified, of whom 88 351 (65.5%) had information regarding patient delay.

### Annual trends by duration of patient delay

**Figure 1:**
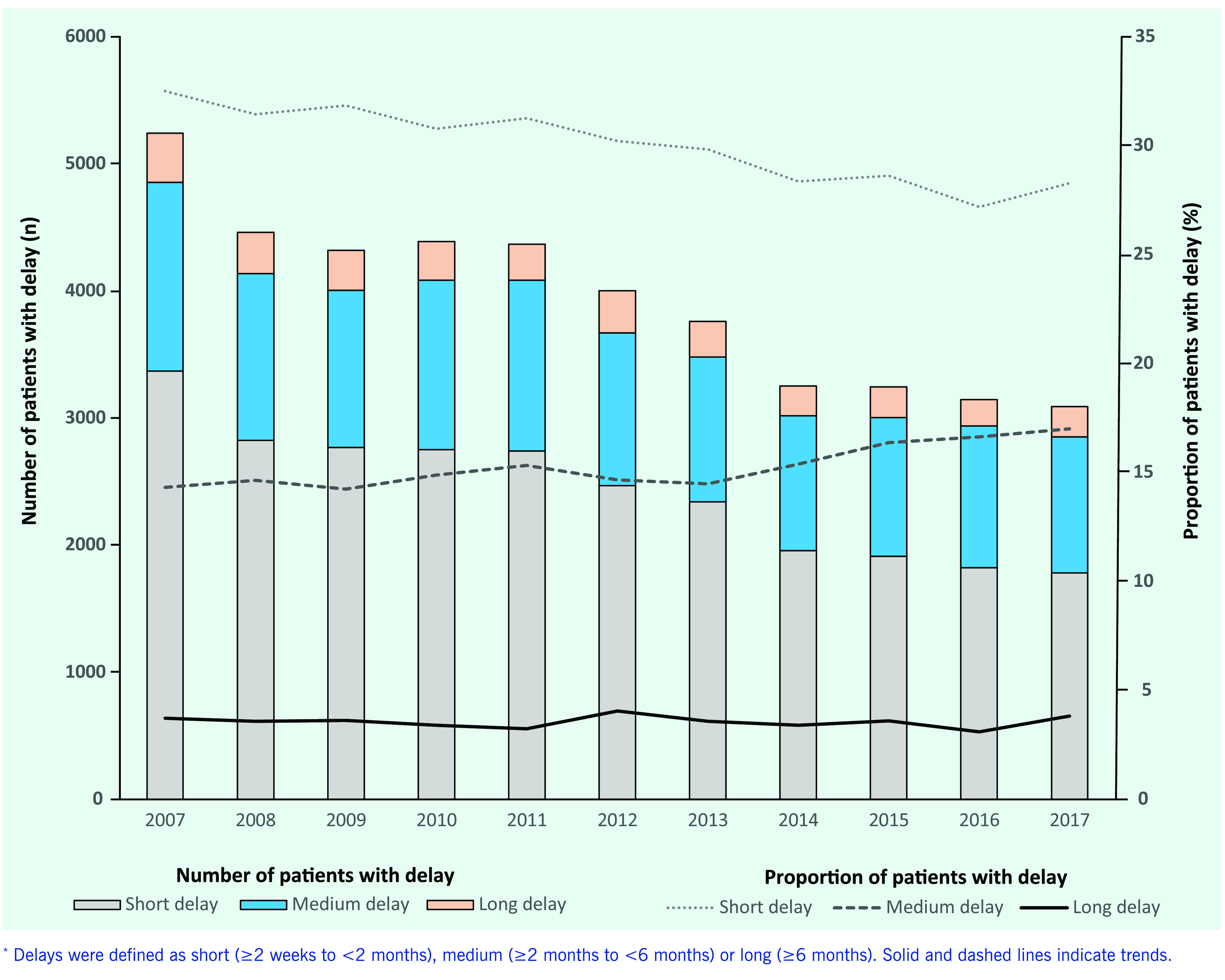
Number and proportion of patients with symptomatic pulmonary tuberculosis categorized by length of delay in seeking care, Japan, 2007–2017^*^

**Figure 2:**
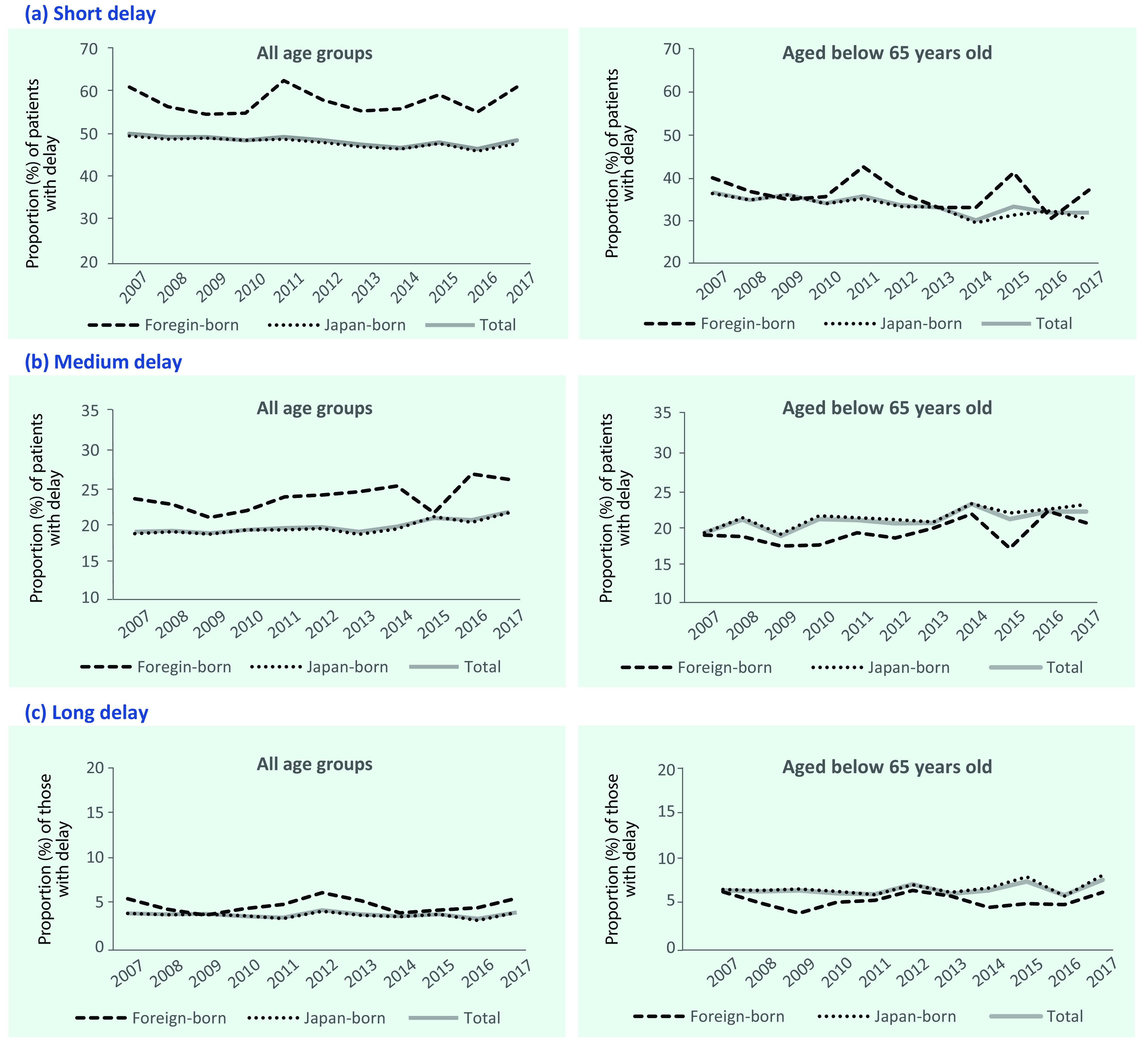
**Annual trends in the proportion of patients with pulmonary tuberculosis and (a) short delay (≥2 weeks to <2 months), (b) medium delay (≥2 months to <6 months) or (c) long delay (≥6 months) in seeking care, by birthplace (born in Japan or outside of Japan) and age group, Japan, 2007–2017**

The annual number of symptomatic PTB patients with any delay decreased from 5242 in 2007 to 3093 in 2017 (**Fig. 1**). The proportion of TB patients with a short delay decreased from 32.5% (3371/10 368) in 2007 to 28.3% (1781/6295) in 2017 (*P* < 0.001). In contrast, the proportions of those with a medium or long delay have been constant or have increased during the study period, from 14.3% (1485/10 368) to 17.0% (1071/6295) (*P* = 0.0015) for those with a medium delay and from 3.7% (386/10 368) to 3.8% (241/6295) for those with a long delay (*P* < 0.001). The annual trends in proportions of those in the three different categories of patient delay for all age groups and for those aged < 65 years, stratified by birthplace (Japan or outside of Japan), are shown in **Fig. 2**.

[**insert** Figure 1]

[**insert** Figure 2]

### Characteristics of patients with delay

The characteristics of PTB patients with and without delay are summarized in **Table 1**. Compared with patients who did not delay seeking treatment, the proportions of men, patients aged 25–54 years, and foreign-born patients were higher in those who delayed seeking treatment. Similarly, the proportions of those receiving public assistance and those without insurance were higher among those with patient delay, and they were higher among those with long delay compared with those with short or medium delay. The proportions of those receiving public assistance among the different types of health insurance status were 11.1% for those with long delay, 8.9% for those with medium delay, and 8.1% for those with short delay. For those who had no insurance among the different types of health insurance status the proportions were 3.1% for those with long delay, 2.4% for those with medium delay, and 1.1% for those with short delay. The proportions of full-time workers (those employed full-time on a mid- to long-term contract), temporary workers (those employed part-time or on a short-term contract) and those with a history of homelessness (those who had been homeless within 1 year of diagnosis) were also higher in those with delay than in those without delay.

**Table 1:** Characteristics of patients with symptomatic pulmonary tuberculosis with and without delay in seeking care, by length of delay, Japan, 2007–2017 (*n* = 88 351)

Category^a^	Patient delay^b^						No delay	
	Short		Medium		Long			
	n	%	n	%	n	%	n	%
**TOTAL**	26 746	100	13 394	100	3 151	100	45 060	100
**Sex**								
Male	17 351	64.9	8 890	66.4	2 195	69.7	28 778	63.9
Female	9 395	35.1	4 504	33.6	956	30.3	16 282	36.1
**Age group (years)**								
0–24	969	3.6	498	3.7	115	3.6	1 209	2.7
25–44	4 102	15.3	2 348	17.5	660	20.9	4 894	10.9
45–64	5 410	20.2	3 475	25.9	1 065	33.8	6 591	14.6
≥ 65	16 265	60.8	7 073	52.8	1 311	41.6	32 366	71.8
**Country of birth**								
Japan-born	24 556	91.8	12 284	91.7	2 864	90.9	41 993	93.2
Foreign-born	1 299	4.9	681	5.1	172	5.5	1 544	3.4
Unknown	891	3.3	429	3.2	115	3.6	1 523	3.4
**Health insurance**								
Covered	23 690	88.6	11 501	85.9	2 587	82.1	40 425	89.7
Public assistance	2 155	8.1	1 195	8.9	349	11.1	3 449	7.7
No insurance	307	1.1	328	2.4	99	3.1	264	0.6
Others	594	2.2	370	2.8	116	3.7	922	2
**Job category**								
Full-time workers	7 036	26.3	3 909	29.2	1 071	34	8 561	19
Temporary workers	1 252	4.7	822	6.1	222	7	1 370	3
Students	442	1.7	238	1.8	41	1.3	574	12.7
Unemployed	16 822	62.9	7 775	58	1 634	51.9	32 770	72.7
Others	767	2.9	390	2.9	107	3.4	1 099	2.4
Unknown	427	9.3	260	1.9	76	2.4	686	1.5
**History of homelessness**								
Yes	440	2.6	343	4	117	6	421	1.5
No	13 886	82.6	6 834	79.8	1 499	76.2	22 798	83.4
Unknown	2 485	14.8	1 383	16.2	350	17.8	4 113	15
**Respiratory symptoms**								
Yes	21 729	81.2	11 417	85.3	2 748	87.2	33 502	74.3
No	5 017	18.8	1 977	14.8	403	12.8	11 558	25.7
**Sputum smear**								
Positive	17 495	65.4	9 909	74	1 205	73.1	25 802	57.3
Negative	9 070	33.9	3 413	25.5	829	26.3	18 950	42.1
Not tested	136	0.5	47	0.4	13	0.4	232	0.5
Unknown	45	0.2	25	0.2	5	0.2	76	0.2

^a^ Public assistance denotes those who were receiving social welfare benefit at the time of diagnosis. Covered indicates those who had health insurance. Full-time workers are those who were employed full-time on a mid- to long-term contract. Temporary workers are those who were employed part-time or on a short-term contract. History of homelessness refers to those who had been homeless within 1 year of diagnosis. Respiratory symptoms included cough, sputum, bloody sputum, and haemoptysis.

^b^ Delays were defined as short (≥ 2 weeks to < 2 months), medium (≥ 2 months to < 6 months) or long (≥ 6 months).

### Risk factors for patient delay

The results of the multinomial regression analysis are summarized in **Table 2**. Male sex was a significant risk factor for long delay (adjusted odds ratio [aOR] = 1.17, *P* < 0.05). Compared with students, being a full-time worker was a risk factor for long delay (aOR = 1.72, *P* < 0.05), while being a temporary worker was a risk factor for any delay (aOR = 1.22, *P* < 0.05 for short delay; aOR = 1.34, *P* < 0.05 for medium delay; and aOR = 1.99, *P* < 0.05 for long delay).

**Table 2:** Results of the multinomial regression analysis for odds ratio (95% confidence interval [CI]) for delays in seeking care among patients with pulmonary tuberculosis, Japan, 2007–2017

Category (reference group)	Patient delay^a^					
	Short		Medium		Long	
	Odds ratio (95% CI)	*P*-value	Odds ratio (95% CI)	*P*-value	Odds ratio (95% CI)	*P*-value
Sex (female)						
Male	1 (0.65 to 1.04)	0.84	1.01 (0.96 to 1.07)	0.68	1.17 (1.06 to 1.31)	< 0.05
Age (25−44 years)						
0–24	0.93 (0.81 to 1.06)	0.27	0.91 (0.78 to 1.07)	0.27	0.95 (0.72 to 1.25)	0.72
45–64	0.99 (0.92 to 1.06)	0.68	1.05 (0.96 to 1.14)	0.28	1.08 (0.94 to 1.24)	0.29
> 65	0.75 (0.69 to 0.80)	< 0.05	0.6 (0.55 to 0.65)	< 0.05	0.38 (0.32 to 0.44)	< 0.05
**Job (students)**						
Full-time workers	1.14 (0.95 to 1.37)	0.17	1.13 (0.91 to 1.42)	0.27	1.72 (1.01 to 2.67)	< 0.05
Temporary workers	1.22 (1.00 to 1.49)	< 0.05	1.34 (1.05 to 1.70)	< 0.05	1.99 (1.25 to 3.16)	< 0.05
Unemployed	0.86 (0.71 to 1.04)	0.12	0.83 (0.66 to 1.04)	0.11	1.3 (0.83 to 2.03)	0.25
Others	1.05 (0.87 to 1.27)	0.65	1.06 (0.84 to 1.34)	0.61	1.83 (1.17 to 2.87)	< 0.05
**Insurance (covered)**						
No insurance	1.63 (1.33 to 2.00)	< 0.05	2.81 (2.29 to 3.46)	< 0.05	2.75 (2.03 to 3.71)	< 0.05
Public assistance	1.06 (0.98 to 1.14)	0.15	1.19 (1.09 to 1.31)	< 0.05	1.36 (1.16 to 1.60)	< 0.05
Others	1.06 (0.89 to 1.26)	0.54	1.27 (1.04 to 1.54)	< 0.05	1.53 (1.12 to 2.08)	< 0.05
**History of homelessness (no history)**						
Yes	1.46 (1.26 to 1.69)	< 0.05	1.73 (1.47 to 2.04)	< 0.05	2.09 (1.63 to 2.67)	< 0.05
**History of treatment (yes)**						
No	1.1 (1.02 to 1.18)	< 0.05	1.29 (1.17 to 1.42)	< 0.05	1.27 (1.05 to 1.52)	< 0.05
**Symptoms (no respiratory symptom)**						
Respiratory symptoms	1.5 (1.42 to 1.58)	< 0.05	1.8 (1.67 to 1.93)	< 0.05	1.89 (1.63 to 2.19)	< 0.05
**Diagnosed with diabetes (No)**						
Yes	1.13 (1.07 to 1.20)	< 0.05	1.23 (1.15 to 1.32)	< 0.05	1.14 (1.00 to 1.30)	< 0.05

^a^ Delays were defined as short (≥ 2 weeks to < 2 months), medium (≥ 2 months to < 6 months) or long (≥ 6 months).

Not having health insurance was a risk factor for all types of delay (aOR = 1.63, *P* < 0.05 for short delay; aOR = 2.81, *P* < 0.05 for medium delay; aOR = 2.75, *P* < 0.05 for long delay) and having a history of homelessness was also a risk factor for all types of delay (aOR = 1.46, *P* < 0.05 for short delay; aOR = 1.73, *P* < 0.05 for medium delay; aOR = 2.09, *P* < 0.05 for long delay). Receiving public assistance was specifically a risk factor for medium and long delays (aOR = 1.19, *P* < 0.05 for medium delay; aOR = 1.36, *P* < 0.05 for long delay) (**Table 2**).

Reporting respiratory symptoms and DM were identified as risk factors for delay. In contrast, being aged ≥ 65 years was a protective factor against all categories of patient delay (aOR = 0.75, *P* < 0.05 for short delay; aOR = 0.60, *P* < 0.05 for medium delay; aOR = 0.38, *P* < 0.05 for long delay).

### Patient delay by health insurance status

**Figure 3:**
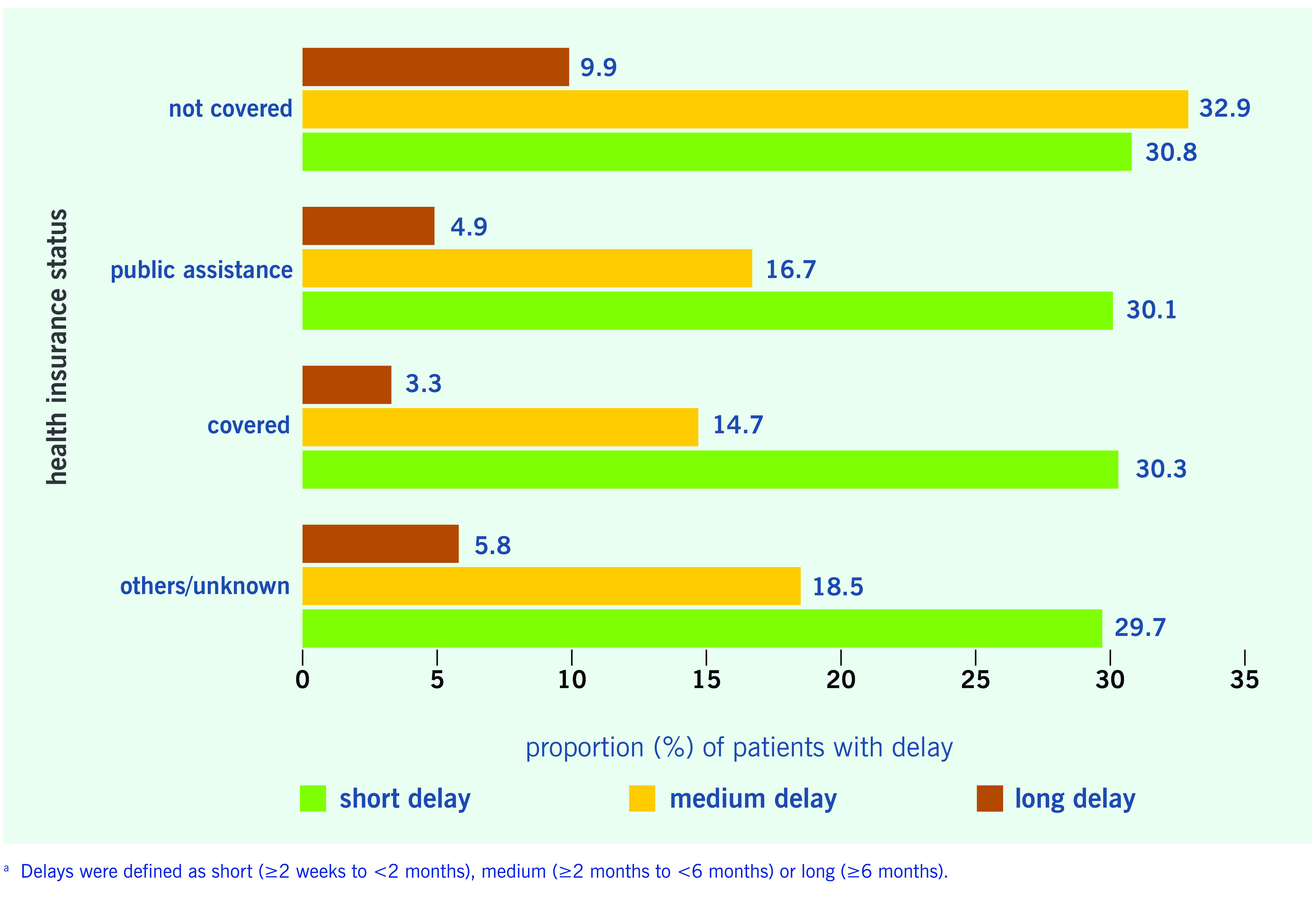
**Proportions of patients with pulmonary tuberculosis with delays in seeking care, by health insurance status, Japan, 2007–2017^a^**

The proportions of patients in each delay category by health insurance status are shown in **Fig. 3**. For all types of health insurance status, the proportions of patients with a short delay were all approximately 30%. However, the proportions of patients who had medium delay or long delay were greater among those without health insurance (32.9% and 9.9%, respectively).

[**insert** Figure 3]

## Discussion

In the absence of a universal definition of patient delay, some international guidelines have stated that all patients with unexplained cough lasting 2 to 3 weeks or longer should be evaluated for TB. (*10*) In fact, definitions of patient delay have varied from 7 to 60 days in previous studies. (*5*, *11*, *12*) Our study is unique in that it is the first detailed study of the trends in and risk factors for patient delay in Japan to use three categories, namely short, medium and long.

We found that the proportions of those with short delay steadily declined during the study period (*P* < 0.001), while the proportions of those with medium or long delay have been constant or even increased (*P* = 0.0015 and *P* < 0.001, respectively), indicating that patient delay remains a major challenge, even in a country where UHC was achieved decades ago.

Although the proportions of those with any delay tended to be higher among foreign-born patients for all age groups and higher for medium or long delay among Japan-born patients younger than 65 years, country of birth was not a significant factor in the multinomial regression analysis.

However, our study identified not having health insurance as one of the key risk factors for patient delay. Previous studies have not agreed on the influence of health insurance, with some suggesting that a lack of health insurance affects patient delay, (*13*) while others have not found this. (*14*) To a certain extent, the inconsistency may reflect country-level differences in health insurance systems and patient eligibility. In Japan, under UHC all residents, including foreign-born persons, are expected to be covered by national health insurance schemes. However, the number of those who are unable or unwilling to pay their premiums has been increasing recently, leading to widening health disparities among people in Japan. (*15*) Those who fail to pay the premium for more than 18 months are disqualified from receiving health insurance benefits; in the event of disqualification, they must pay the full cost of medical services after each visit to a medical facility. (*16*) According to a report from the Japan Medical Practitioner’s Association, the frequency of outpatient clinic utilization was significantly lower among those without health insurance – that is, it was one seventieth of those with health insurance. (*17*) Such a study strongly indicates that not having health insurance is a serious barrier to accessing health care; our study found that TB patients are not an exception.

Receiving public assistance was another risk factor for patient delay. In Japan, public assistance is available to low-income households that are not capable of paying health insurance premiums − such as households in which people have a long-term illness or disability or are headed by a single parent – and those receiving social welfare are totally exempt from health insurance premiums as well as out-of-pocket payments. Indeed, a recent report by a governmental working group on social welfare described a higher frequency of hospital visits among those receiving public assistance compared with those covered under other health insurance schemes. (*18*) In contrast, several studies have suggested that those receiving public assistance had a low participation rate in community health screenings (*19*) and a higher smoking rate. (*20*, *21*) As smoking is often perceived as being associated with non-specific “smoker’s cough,” it has been identified by several studies as a risk factor for patient delay among TB patients. (*22*, *23*) In other words, there may be confounding effects between smoking and receiving public assistance. Another study has suggested that even among those receiving public assistance, participation rates in community health checks were lower among those who had been receiving public assistance for longer than 5 years and among those who had not had any health insurance before receiving public assistance. (*24*) Further studies are necessary to explore the health-seeking behaviour of TB patients who are receiving public assistance.

Our results also indicated that being a temporary worker and having a history of homelessness are risk factors for patient delay, consistent with previous studies from Japan. (*25*-*27*) In fact, the populations of temporary laborers and homeless people overlap, as temporary laborers may lack permanent addresses and, thus, may be classified as homeless, and people who are truly homeless often earn income from ad hoc jobs, such as construction and cleaning. The fear of losing income or a job as a result of taking time off from work to seek health care or being diagnosed with an illness are major barriers to seeking health care among people with precarious job situations. (*26*)

TB control activities specifically targeting homeless people have been in place in several urban areas in Japan, including mobile screening by chest X-ray, free screening at accommodation for people seeking asylum and screening upon moving into affordable housing. (*28*, *29*) Yet various studies continue to indicate that homeless people have limited access to health care for a variety of sociopsychological and economic reasons. (*30*, *31*) One of the major issues in TB control among homeless people is the increasing diversification of the profile of so-called homeless people, a label that can include elderly people without night-time shelter, middle-aged men living on day-to-day jobs and sleeping in internet cafes, and teenagers who cannot live with their parents and so move from one friend’s house to another. (*32*) Traditional outreach services, such as mobile screening on streets and in shelters, may not reach a significant proportion of people who are classified as homeless.

Two distinct factors were associated with long delays, namely being male and being a full-time worker. A study from Osaka city, Japan, similarly reported that TB patients with a job were more likely to delay seeking care compared with those without a job. (*26*) In the same study, the authors compared the reasons for not seeking care promptly among those who delayed seeking care and those who did not and revealed that the proportion of those who had been too busy with work and were unable to take time off was significantly higher among those who delayed seeking care. In another study that examined participation rates for general medical check-ups, the authors similarly reported that compared with those without jobs, a higher proportion of those with jobs did not participate in medical check-ups. (*33*)

In our study, being male was an independent risk factor for long delay. However, contrary to our findings, a systematic review of delay among TB patients in Asia reported that being male was significantly associated with shorter patient delay. (*34*) TB prevalence is generally higher among men, possibly leading to a greater awareness of TB and subsequent health-care-seeking behaviour among men compared with women; women also may face greater financial and cultural barriers to seeking care. However, a different study concluded that the higher prevalence of TB among men was precisely due to a longer delay before diagnosis. (*35*) Further analyses should explore the inconsistencies in these findings; however, several studies on health-seeking behaviour in Japan have shown that men are generally less motivated to participate in medical check-ups (*36*) and community screening opportunities. (*37*)

Being diagnosed with TB for the first time (i.e. being a new case), having respiratory symptoms and having DM as a co-morbid condition were also identified as risk factors for patient delay. A new case may be considered to be a proxy for a lack of or limited knowledge of TB, which has been reported to hinder patients from accessing care; several studies have shown that individuals with a previous history of TB were more likely to seek care earlier because of their previous exposure to the disease and TB-related services and also, potentially, their increased knowledge. (*38*, *39*) Conclusions from previous studies on the association between patient delay and symptoms have been contradictory: while some have shown that patients with symptoms tended to seek care early, (*40*, *41*) others have found the opposite, which was attributed to the possibility that patients did not consider their symptoms serious enough to need health care. (*42*) In our study, a similar explanation may be possible. Because our definition of respiratory symptoms included non-severe and general symptoms, such as cough, it is possible that patients misjudged their illness. As for DM, a previous study in Japan on DM among patients with PTB also similarly reported a longer delay among those with DM. (*43*) It has been previously reported that, in general, patients with DM are less willing to seek medical care, (*44*) and the authors suggested that this may have also affected delays in seeking TB care.

Finally, being aged 65 years or older was identified as a protective factor against patient delay. Similar results have been reported from other countries, including Norway (*45*) and Italy. (*46*) It has been suggested that elderly patients often have coexisting illnesses and thus routinely visit hospitals, thereby increasing the likelihood of seeking care when they have TB-related symptoms.

Our study has several limitations. First, patients for whom there was no information regarding their delay in seeking care were excluded from our analyses. Some of these patients eventually died as a result of an extremely long delay, but because the patients were already too sick at the time of diagnosis, public health nurses were unable to interview them and collect the data that allow us to calculate the length of delay. In the surveillance data, approximately one third of patients had no information on delay, and, as such, it is possible that our results may underestimate the real magnitude of patient delay. Second, because we analysed data from the JTBS system, other potential risk factors, such as smoking, could not be considered. The results of our study should be interpreted along with results from local studies that have used local data held by public health centres. Last, as the onset date of symptoms was self-reported by patients, it could have been affected by recall bias.

Interventions to prevent patient delay should be designed to address specific risk factors. Providing waivers for out-of-pocket expenses under certain conditions, especially for those without health insurance, and providing a sickness allowance for those with precarious work situations would potentially improve access. Furthermore, it is equally important to implement more general interventions to improve the working environment to allow workers to take leave to seek medical services without feeling ashamed or guilty. Actions taken within the health sector alone cannot achieve and maintain UHC, and increasing effort is required to build the capacity for multisectoral approaches. Community health screening tailored to those who do not have health insurance or are receiving social welfare could help early case detection, especially if undertaken in collaboration with municipal health authorities. Activities to increase the awareness of TB symptoms should also be strengthened, especially among groups likely to have longer delays seeking care, particularly men and full-time workers.

## Conclusions

In spite of the implementation of UHC decades ago in Japan, a detailed analysis of surveillance data has revealed that patient-led delays in TB diagnosis are still a major challenge. This study’s results identified various risk factors, many of which could be mitigated by implementing appropriate social protection measures, indicating the shortcomings of UHC in Japan and the need for continued effort to ensure that no one is left behind.
